# Belatacept in Kidney Transplantation: What Are the True Benefits? A Systematic Review

**DOI:** 10.3389/fmed.2022.942665

**Published:** 2022-07-14

**Authors:** Yannis Lombardi, Hélène François

**Affiliations:** ^1^Soins Intensifs Néphrologiques et Rein Aigu, Hôpital Tenon, Assistance Publique-Hôpitaux de Paris, 4 rue de la Chine, Paris, France; ^2^Sorbonne Université, Paris, France; ^3^INSERM UMR_S 1155, Paris, France

**Keywords:** belatacept, kidney transplantation, immunosuppressive therapy, maintenance therapy, calcineurin avoidance, avoidance (withdrawal), CNI toxicity, costimulation blockade

## Abstract

The current gold standard to prevent allograft rejection for maintenance immunosuppression in kidney transplantation currently consists in glucocorticoids, an antiproliferative agent and a calcineurin inhibitor (CNI), with better outcome for tacrolimus than cyclosporin. Although, CNI drastically improved early graft survival, so far, CNI have failed to significantly improve long-term survival mainly because of nephrotoxicity. In addition, CNI carry several other side effects such as an increased risk for cardiovascular events and for diabetes mellitus. Therefore, seeking alternatives to CNI remains of paramount importance in kidney transplantation. Belatacept is a fusion protein composed of the human IgG1 Fc fragment linked to the modified extracellular domain of cytotoxic T lymphocyte–associated antigen 4. In kidney transplant recipients, pivotal phase III randomized studies suggested clinical benefits of belatacept as an initial maintenance regimen, as compared with cyclosporine, mainly on kidney function. Recently, a randomized study also suggested a clinical benefit on renal function of a conversion from a CNI-based to a belatacept-based maintenance regimen in patients. However, conversion from CNIs to belatacept is probably associated with an increased risk of biopsy-proven acute rejection and should prompt close clinical surveillance. On the other hand, other studies suggest a decrease in *de novo* humoral transplant immunization. Belatacept is probably associated with an increase in both risk and severity of some infectious diseases, including EBV-linked post-transplantation lymphoproliferative disorders, and with a decreased response to vaccines. Most studies on belatacept are observational, retrospective, and non-comparative. Consequently, high-quality data about the safety and efficacy profile of belatacept, as compared with the current gold standard for maintenance regimens (tacrolimus-based), is uncertain. Our review will therefore focus on the most recent published data aiming at evaluating the evidence-based or the “true” benefits and risks of belatacept-based regimens in kidney transplantation.

## Introduction

The current gold standard to prevent allograft rejection in kidney transplantation currently consists in a maintenance treatment based on glucocorticoids, an antiproliferative agent and a calcineurin inhibitor (CNI) (1). Among calcineurin inhibitors, tacrolimus is the current gold standard due to better outcomes as compared to cyclosporin A (1). Indeed, CNI drastically improved early graft survival but, so far, have failed to improve significantly long-term survival mainly because of nephrotoxicity. In addition, CNI carry several other side effects such as an increased risk for cardiovascular events and for diabetes mellitus (2). Therefore, seeking alternatives to CNI remains of paramount importance in kidney transplantation.

Belatacept was designed as an alternative to calcineurin inhibitors-based regimens to prevent rejection–and consequently, graft loss–in recipients of kidney allografts. Belatacept is a recombinant immunoglobulin fusion protein, combining the modified extracellular B7-binding domain of Cytotoxic T-Lymphocyte-Associated protein 4 (CTLA4) with the constant fragment portion (Fc) of IgG1 (3). Due to a high affinity with CD80 (B7-1) and CD86 (B7-2), molecules expressed on Antigen Presenting Cells, belatacept acts as a highly potent costimulation inhibitor, preventing CD28-mediated T-cell activation (3).

Since belatacept appeared effective in preventing allograft rejection in non-human models of kidney transplantation without the burden of nephrotoxicity (3), subsequent clinical studies were led. Belatacept obtained US Food and Drug Administration's and European Medicines Agency's approval as an alternative for CNI in *de novo* kidney transplant recipients (KTRs) in 2011 (4), although initial trials were led against cyclosporin. Yet, in 2016, only 3.11% of *de novo* KTRs the United States received belatacept for initial maintenance therapy (5). Similarly, its use in France and in many countries in Europe has been limited because meta-analysis have failed to demonstrate significant benefits for long term graft survival compared to tacrolimus (6).

In addition, despite its lack of nephrotoxicity and a better renal graft function several questions remain that may hamper its use in clinical practice such as the risk of acute rejection, PTLD and infection.

In view of the recent published randomized trials that were led against tacrolimus (7, 8), we will hereafter review the benefits and risks of using belatacept in kidney transplantation, to provide an up to date and unbiased evaluation of belatacept use in kidney transplantation. To this end, we conducted a systematic review of the literature. Our focus will be on comparative original studies—and mainly Randomized Controlled Trials (RCTs)—studying the impact, on clinically pertinent outcomes, of using belatacept instead of CNI. We will also briefly review other studies.

## Systematic Review of the Litterature

**Figure 1:**
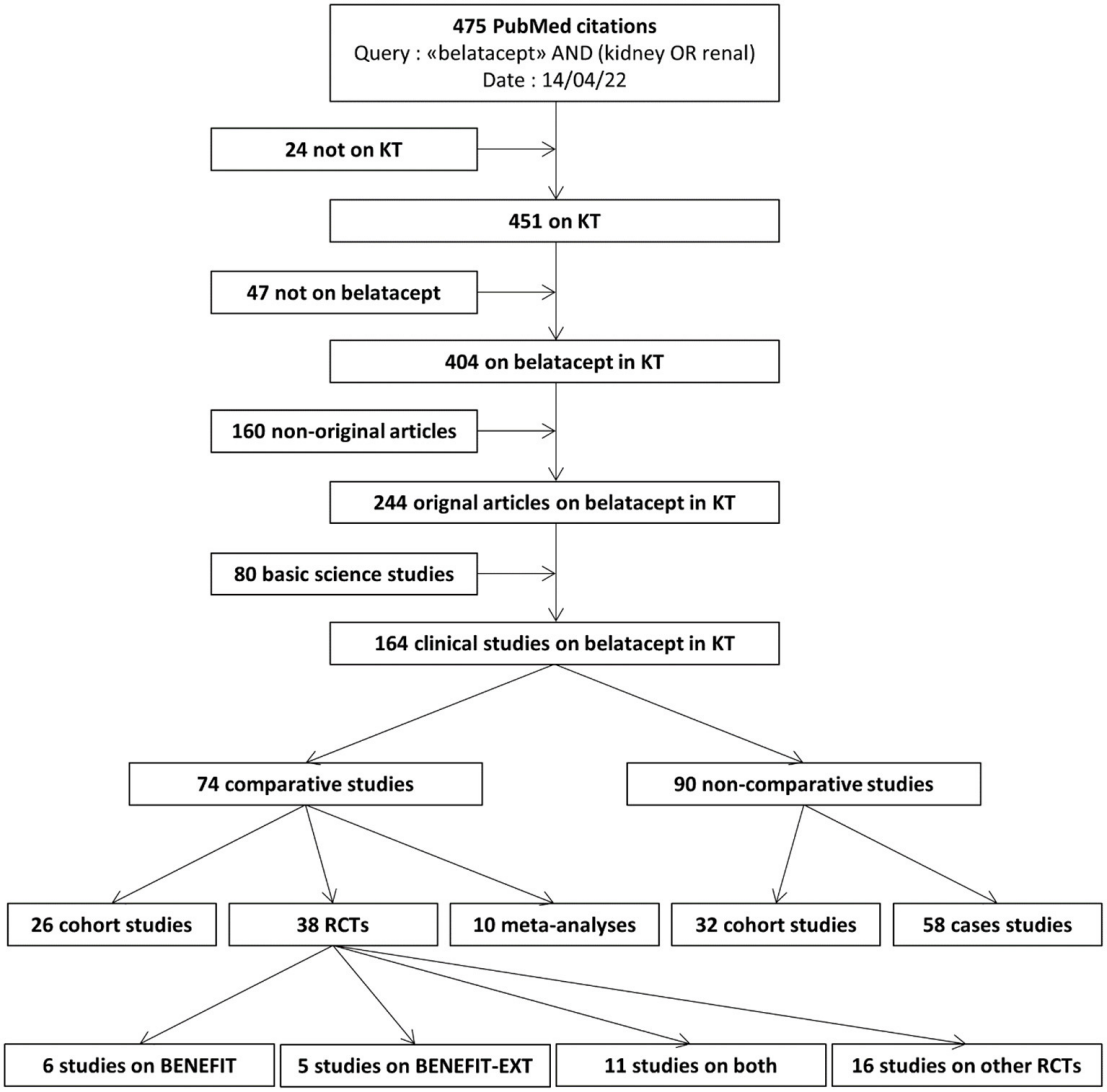
Systematic review of the literature on belatacept in kidney transplantation. KT: kidney transplantation. RCT: randomized controlled trial.

We performed a systematic review of the current medical literature (Figure 1). We searched NCBI's PubMed database on 14/04/2022 using the query [“belatacept” AND (kidney OR renal)] and identified 475 citations. We assessed all corresponding abstracts.

We retrieved 404 articles on belatacept in kidney transplantation, among which 160/404 (39.6%) were not original studies (i.e., reviews, experts' opinions, comments, responses, etc..).

We retrieved 80 basic science studies and 164 clinical studies. Among clinical studies, 90/164 (54.9%) were non-comparative studies, meaning that no comparison was made between belatacept and other treatments.

## Randomized Clinical Trials Comparing CNI- and Belatacept-Based Regimens

We retrieved 38 published articles on RCTs comparing belatacept with at least one other treatment. Among these, 22/38 (57.9%) concerned Belatacept Evaluation of Nephroprotection and Efficacy as First-line Immunosuppression Trial (BENEFIT) trials—either BENEFIT, BENEFIT-Extended Criteria Donors (BENEFIT-EXT), or both.

We will consider, for each RCT, the publication describing the longest follow-up for each RCT in the intention-to-treat population. In general, unless there is a significant contribution, we will not discuss results from publications describing short-term analyses, *post-hoc* analyses, subgroup analyses or meta-analyses of these RCTs.

Overall, 13 distinct RCTs were identified, 11 of which were trials directly comparing CNI- and belatacept-based regimens (Table 1). One study was not considered since it investigated the effect of belatacept to prevent humoral sensitization in patients with failed grafts. One study compared two belatacept regimens (every 4 weeks vs. every 8 weeks); its results are also reported in Table 1.

**Table 1:** Summary of randomized controlled trials evaluating belatacept in kidney transplant recipients.

	**Comparator**	**Intervention**	**Nb. of patients**	**Setting**	**Follow–up**	**Death**	**Graft failure**	**Death or graft failure**	**Rejections**	**CV events**	**Infections**	**Cancers**
**Belatacept vs. CNI, for** ***de novo*** **KT recipients**												
Vincenti et al. (9)	CsA (C_0_: 150–300 mg/l until M1, then 100–250)	First randomization: LI: 10 mg/kg, 6 inj./3 months MI: 10 mg/kg, 11 inj./6 months Second randomization: 4w: 5 mg/kg every 4w 8w: 5 mg/kg every 8w	(Total nb: 218) CsA: 73 LI: 71 MI: 74 CsA:71 4w: 62 8w: 60	Basiliximab for induction, steroids + MYC for maintenance	10 years	(Total nb: 15) CsA: 5/73 LI: 2/71 MI: 8/74	(Total nb: 8) CsA: 3/73 LI: 1/71 MI: 4/71	LI vs. CsA: HR 0.95 [0.38–2.36] **MI vs. CsA:** **HR 0.24 [0.24–0.91]** 4w vs. CsA: HR 0.55 [0.17–1.73] 8w vs. CsA: HR 0.52 [0.16–1.74]	LI vs. CsA: HR 1.61 [0.85–3.05] MI vs. CsA: HR 0.95 [0.47–1.92] 4w vs. CsA: HR 1.06 [0.35–3.17] 8w vs. CsA: HR 2.00 [0.75–5.35]	(Death from CV cause) CsA: 2/73 LI: 1/71 MI: 1/71	(Serious events) CsA: 15.0/100py LI: 6.7/100py MI: 10.4/100py CsA: 16.7/100py 4w: 6.0/100py 8w: 10.4/100py	CsA: 3.0/100py LI: 2.5/100py MI: 10.4/100py CsA: 3.3/100py 4w: 2.8/100py 8w: 3.3/100py
Vincenti et al. (10) BENEFIT	CsA (C_0_: 150–300 mg/l until M1, then 100–250)	Bela LI then 4w Bela MI then 4w	(Total nb: 666) CsA: 221 LI: 226 MI: 219	SCD Basiliximab for induction, steroids + MYC for maintenance	7 years	(Total nb: 58) LI vs. CsA: HR 0.55 [0.30–1.04] MI vs. CsA: HR 0.62 [0.33–1.14]	(Total nb: 38) LI vs. CsA: 0.59 [0.28–1.25] MI vs. CsA: 0.56 [0.25–1.21]	**LI vs. CsA:** **HR 0.57 [0.35–0.94]** **MI vs. CsA:** **HR 0.57 [0.35–0.95]**	CsA: 11.4% LI: 18.3% MI: 24.4%	(Death from CV cause) CsA: 11/221 LI: 6/226 MI: 6/219 (Serious cardiac+vascular events) CsA: 2.0+1.8/100py LI: 1.4+1.5/100py MI: 2.2+2.9/100py	(Serious events) CsA: 13.3/100py LI: 10.7/100py MI: 10.6/100py	CsA: 2.6/100py LI: 1.8/100py MI: 2.1/100py
Durrbach et al. (11) BENEFIT–EXT	CsA (C_0_: 150–300 mg/l until M1, then 100–250)	Bela LI then 4w Bela MI then 4w	(Total nb: 542) CsA: 184 LI: 175 MI: 183	ECD Basiliximab for induction, steroids + MYC for maintenance	7 years	(Total nb: 102) LI vs. CsA: HR 0.78 [0.45–1.35] MI vs. CsA: HR 0.70 [0.40–1.29]	(Total nb: 73) LI vs. CsA: 0.78 [0.45–1.35] MI vs. CsA: 0.70 [0.40–1.23]	LI vs. CsA: HR 0.93 [0.63–1.36] MI vs. CsA: HR 0.92 [0.63–1.34]	LI vs. CsA: HR 1.15 [0.70–1.90] MI vs. CsA: HR 1.22 [0.75–2.00]	(Death from CV cause) CsA: 8/184 LI: 12/175 MI: 12/183 (Serious events) CsA: 5.2/100py LI: 4.1/100py MI: 5.2/100py	(Serious events) CsA: 20.3/100py LI: 16.5/100py MI: 22.7/100py	CsA: 3.6/100py LI: 3.2/100py MI: 3.8/100py
Ferguson et al. (12)	Tac/MYC (C_0_: 8–12 ng/ml until M1, then 5–10)	Bela/MYC Bela/Siro	(Total nb: 89) Tac/MYC: 30 Bela/MYC: 33 Bela/Siro: 26	rATG for induction No steroids for maintenance	1 year	(Total nb: 1) Tac/MYC: 0/30 Bela/MYC: 1/33 Bela/Siro: 0/26	(Total nb: 3) Tac/MYC: 0/30 Bela/MYC: 1/33 Bela/Siro: 2/26	Tac/MYC: 0/30 Bela/MYC: 2/33 Bela/Siro: 2/26	Tac/MYC: 1/30 Bela/MYC: 5/33 Bela/Siro: 1/26	–	Tac/MYC: 5/30 Bela/MYC: 7/33 Bela/Siro: 4/26	Tac/MYC: 1/30 Bela/MYC: 0/33 Bela/Siro: 1/26
de Graav et al. (13)	Tac (C_0_: 10–15 ng/ml until S2, then 8–12 until M1, then 5–10)	Bela	(Total nb: 40) Tac: 20 Bela: 20	Basiliximab for induction, steroids + MYC for maintenance	1 year	(Total nb: 1) Tac: 1/20 Bela: 0/20	(Total nb: 3) Tac: 0/20 Bela: 3/20	Tac: 1/20 Bela: 3/20	Tac: 2/20 Bela: 11/20	Tac: 1.20/100py Bela: 0.95/100py	Tac: 1.90/100py Bela: 2.25/100py	Tac: 0/100py Bela: 0/100py
Newell et al. (14) CTOT−10	Alem/Tac (C_0_: 8–12 ng/ml until M6, then 5–10)	Alem/Bela Bas/Tac/Bela: tacrolimus withdrawal in 3 months	(Total nb: 19) Alem/Tac: 6 Alem/Bela: 6 Bas/Tac/Bela: 7	No steroids for maintenance	1 year	(Total nb: 1) Alem/Tac: 1/6 Alem/Bela: 0/6 Bas/Tac/Bela: 0/7	(Total nb: 4) Alem/Tac: 1/6 Alem/Bela: 3/6 Bas/Tac/Bela: 0/7	Alem/Tac: 2/6 Alem/Bela: 3/6 Bas/Tac/Bela: 0/7	Alem/Tac: 3/6 Alem/Bela: 2/6 Bas/Tac/Bela: 5/7	–	–	–
Stock et al. (15) CTOT−15	MYC/Tac (C_0_: 8–12 ng/ml until M6, then 5–8)	MYC/Tac/Bela: tacrolimus withdrawal, if possible, in 10 months	(Total nb: 43) MYC/Tac: 21 MYC/Tac/Bela: 22	Combined kidney and pancreas transplantation rATG for induction No steroids for maintenance	1 year	(Total nb: 1) MYC/Tac: 0/21 MYC/Tac/Bela: 1/22	(Total nb: 0)	MYC/Tac: 0/21 MYC/Tac/Bela: 1/22	(Treated episodes) MYC/Tac: 4/21 MYC/Tac/Bela: 4/22	–	MYC/Tac: 15/21 MYC/Tac/Bela: 19/22	–
Mannon et al. (16) CTOT−16	rATG/MYC/Tac (C_0_: 8–12 ng/ml until M6, then 5–8)	rATG/MYC/Bela Bas/Tac/MYC/ Bela: tacrolimus withdrawal in 3 months	(Total nb: 68) rATG/MYC/Tac: 29 rATG/MYC/Bela: 29 Bas/Tac/MYC/ Bela: 10	No steroids for maintenance	1 year	(Total nb: 2) rATG/MYC/Tac: 2/29 rATG/MYC/Bela: 0/29 Bas/Tac/MYC/Bela: 0/11	(Total nb: 0)	rATG/MYC/Tac: 2/29 rATG/MYC/Bela: 0/29 Bas/Tac/MYC/ Bela: 0/11	(Treated episodes) rATG/MYC/Tac: 7/29 rATG/MYC/Bela: 14/29 Bas/Tac/MYC/Bela: 4/11	–	rATG/MYC/Tac: 14/29 rATG/MYC/Bela: 16/29 Bas/Tac/MYC/Bela: 3/11	–
Kaufman et al. (17) BEST	rATG/Tac (C_0_: 8–12 ng/ml until M1, then 5–10)	rATG/Bela Alem/Bela	(Total nb: 333) rATG/Tac: 105 rATG/Bela: 104 Alem/Bela: 107	No steroids for maintenance	2 years	(Total nb: 7) rATG/Tac: 1/105 rATG/Bela: 4/104 Alem/Bela: 2/107	(Total nb: 2) rATG/Tac: 1/105 rATG/Bela: 1/104 Alem/Bela: 0/107	rATG/Tac: 2/105 rATG/Bela: 5/104 Alem/Bela: 2/107	rATG/Tac: 7/105 rATG/Bela: 26/104 Alem/Bela: 20/107	(Serious events) rATG/Tac: 3/105 rATG/Bela: 10/104 Alem/Bela: 1/107	(Serious events) rATG/Tac: 22/105 rATG/Bela: 24/104 Alem/Bela: 24/107	rATG/Tac: 7/105 rATG/Bela: 6/104 Alem/Bela: 7/107
**Belatacept vs. CNI, for stable KT recipients already on CNI**												
Grinyo et al. (7)	CNI (CsA or Tac)	Bela: 5 mg/kg 5 inj./2 months, then every 4w	(Total nb: 173) CNI: 89 Bela: 84	6–36 months after KT eGFR 35–75 ml/min	3 years	(Total nb: 2) CNI: 1/89 Bela: 1/84	(Total nb: 2) CNI: 1/89 Bela: 1/84	CNI: 2/89 Bela: 2/84	CNI: 3/89 Bela: 7/84	–	(Serious events) CNI: 10.2/100py Bela: 9.3/100py	CNI: 3.4/100py Bela: 3.0/100py
Budde et al. (8)	CNI (CsA or Tac)	Bela	(Total nb: 666) CNI: 223 Bela: 223	6–60 months after KT eGFR 30–75 ml/min	2 years	(Total nb: 8) CNI: 4/223 Bela: 4/223	(Total nb: 2) CNI: 2/223 Bela: 0/223	CNI: 6/223 Bela: 4/223	CNI: 9/223 Bela: 18/223	(Death from CV cause) CNI: 1/223 Bela: 3/223	(Serious events) CNI: 44/222 Bela: 37/221	CNI: 12/222 Bela: 18/221
**Comparison of belatacept regimens, for stable KT recipients already on belatacept**												
Badell et al. (18)	Bela 4w	Bela 8w	(Total nb: 163) 4w: 82 8w: 81	>12 months after KT eGFR >35 ml/min	1 year	(Total nb: 2) 4w: 2/82 8w: 0/81	(Total nb: 0) 4w: 0/82 8w: 0/81	4w: 2/82 8w: 0/81	4w: 2/82 8w: 5/81	–	(Any event) 4w: 24/82 8w: 23/81	4w: 1/82 8w: 4/81

*When several articles were published on the same trial, only the one with the longest follow-up time was considered. Statistically significant differences are highlighted in bold. Bela: belatacept. LI, less intensive: MI, more intensive; CsA, cyclosporin A; Tac, tacrolimus; CNI, calcineurin inhibitors; KT, kidney transplant; MYC, mycophenolate mofetil or mycophenolic acid; rATG, rabbit antithymocyte globulin; Alem, alemtuzumab; Bas, basiliximab; HR, hazard ratio; Py, patient-year*.

Among those 11 RCTs, 9/11 (81.8%) evaluated belatacept in *de novo* kidney transplant recipients (KTRs), and 2/11 evaluated it when started in stable kidney transplant recipients already receiving CNI. Studies in *de novo* KTRs included 2018 patients, 1208/2018 (59.8%) of whom were in BENEFIT and BENEFIT-EXT trials.

Control groups included 1001 patients who received CNI: in 3 trials, 478 patients received only cyclosporin A; in 6 trials, 211 patients received only tacrolimus; in 2 trials, 312 patients received either cyclosporin A or tacrolimus.

Standard regimen for *de novo* KTRs (called “less intensive”), used in all studies, consists in i.v. belatacept 10 mg/kg for 5 injections in 84 days (one every 2 weeks), then 5 mg/kg every month subsequently. This regimen is US Food and Drug Administration- and European Medicines Agency-approved. An alternative regimen (called “more intensive”) consists in i.v. belatacept 10 mg/kg for 11 injections in 6 months, then 5 mg/kg every month subsequently, and was evaluated in three studies.

### Death With a Functioning Graft

Two studies using cyclosporin A as a comparator (BENEFIT and BENEFIT-EXT), and none using tacrolimus, appeared to have sufficient power to perform statistical comparisons for this outcome. In these, no significant difference in the risk of death with a functioning graft were observed between patients receiving belatacept or cyclosporin A, regardless of belatacept dose.

### Death-Censored Loss of Allograft Function

Here also, only BENEFIT trials appeared to have sufficient power for this outcome. In those, no significant difference in the risk of loss of allograft function were observed between patients receiving belatacept or cyclosporin A, regardless of belatacept dosage.

### Graft Loss (Death or Loss of Allograft Function)

Three studies using cyclosporin A as a comparator, and none using tacrolimus, appeared to have sufficient power for this outcome [BENEFIT, BENEFIT-EXT, and the initial phase II study, whose final results were published by Vincenti et al. (9)].

In BENEFIT, belatacept at a standard dose (“less intensive”) was associated with a significant decrease in the risk of graft loss, with a hazard ratio of 0.57 [95% CI: 0.35-0.94] during a 7-year follow-up, when compared with cyclosporin A, in patients receiving a kidney from a Standard Criteria Donor (SCD). All patients were treated by basiliximab at induction, and glucocorticoids and either mycophenolic acid or mycophenolate mofetil for maintenance.

In BENEFIT and the phase II trial, belatacept at a higher dose (“more intensive”) was associated with a significant decrease in the risk of graft loss when compared with cyclosporin A.

In BENEFIT-EXT, in KTRs receiving a kidney from an Expanded Criteria Donora (ECD), regardless of the dose, belatacept was not associated with a significant difference in the risk of graft loss in comparison with cyclosporin A.

### Rejection

In 9 trials out of 11, belatacept was associated with a higher rate of rejection compared with CNI. Statistical comparisons were not systematically performed in these studies but, since this difference is consistently observed across trials in various settings, it most likely reflects a true difference.

Based on data from BENEFIT studies, we can estimate that, in *de novo* KTRs receiving an induction with basiliximab, the risk for biopsy-proven rejection within 7 years following KT using a kidney from an SCD is approximatively 15% higher with belatacept than with cyclosporin A; and 60% higher when using a kidney from an ECD.

Based on data from two RCTs (7, 8), we can estimate that in stable KTRs receiving CNI for more than 6 months, the risk for biopsy-proven rejection within 2–3 years following a switch from CNI to belatacept is increased by approximatively 100-150%, compared to remaining on CNI.

Overall, most rejection episodes occurred within a year following KT (in de novo KTRs) or switch (in stable KTRs).

### Cardiovascular Events

No study had sufficient power to detect a significant difference in death from cardiovascular cause, and no study presented a survival analysis for this outcome.

In BENEFIT studies in general, observed rates of serious cardiovascular events were lower in patients treated with “less intensive” belatacept compared with CsA. In BENEFIT-EXT especially, a trial in which the absolute number of events is high (elderly patients with comorbidities), the rate of serious cardiovascular events was 5.2 per 100 patient-year with cyclosporin A and 4.1 per 100 patient-year with belatacept (relative risk reduction: 21%; absolute risk reduction:−1.1 per 100 patient-year; number needed to treat to avoid one serious cardiovascular event each year: 91 patients).

In the study by Kaufman et al. (17), in 209 patients that received rabbit antithymocyte globulin (rATG) in induction and a steroids-free regimen for maintenance that were followed for 2 years, the rate of serious cardiovascular events was 3.4 times higher in patients treated with belatacept than with tacrolimus (2.8% of patients on tacrolimus vs. 9.6% on belatacept). This difference was not observed when belatacept-treated patients received alemtuzumab instead of rATG.

### Infectious Events

In the initial phase II study, then in BENEFIT and BENEFIT-EXT, a notable decrease in the risk of serious infectious events was noted in patients treated with belatacept, compared with cyclosporin A. For instance, in BENEFIT, the risk of serious infection was 13.3 per 100 patient-year on cyclosporin A and 10.7 per 100 patient-year on belatacept (relative risk reduction: 19.6%; absolute risk reduction:−2.6 per 100 patient-year; number needed to treat to avoid one serious infection each year: 38 patients).

Subsequent studies, that used tacrolimus as the main comparator, did not find such a high decrease in the risk of infection. For instance, in the study by Kaufman et al., the risk of serious infection during the 2-year follow-up was 22/105 (20.9%) on tacrolimus and 24/104 (23.1%) on belatacept. In the study by Budde et al., the risk of serious infection during the 2-year follow-up was 44/222 (19.8%) on CNI and 37/221 (16.7%) on belatacept.

No study was powered to detect significant differences in specific types of infection (e.g., opportunistic infection, CMV disease, BK virus nephropathy, EBV-induced post-transplantation lympoproliferative disorder (PTLD), etc..). However, in the initial phase II study, three patients randomized to receive belatacept developed EBV-induced PTLD, vs. none among cyclosporin A-treated controls. In two of them, the disease was the consequence of a primo infection. In BENEFIT, among EBV-seronegative patients, 5/369 developed EBV-induced PTLD on belatacept, vs. 0/184 on cyclosporin A. Consequently, due to an increase in risk for PTLD in case of EBV primo infection, belatacept is contraindicated for EBV-seronegative patients.

### Cancers

The same limits about statistical power apply for cancers. In BENEFIT-EXT, a trial in which elderly patients were included and, consequently, in which the absolute risk for cancers was high, there was no obvious difference in the risk for cancer between belatacept- and cyclosporin A-treated patients (3.6/100 patient-year on cyclosporin A vs. 3.2 on belatacept, during a 7-year follow-up). In the study by Budde et al. (8), during a 2-year follow-up, 5.4% of patients in the CNI group (90% of whom received tacrolimus) developed a cancer, vs. 8.1% in the belatacept group.

No study had sufficient power to detect differences on the risk for specific cancers (e.g., non-skin cancers).

### Estimated Glomerular Filtration Rate and Donor Specific Antibodies

In most studies, belatacept was associated with an increase in estimated glomerular filtration rate (eGFR) compared to CNI. This is likely a consequence of differential renal hemodynamic effects between both drugs. In BENEFIT, after a 7-year follow-up, mean eGFR increased from 66 to 72.1 ml/min/1.73 m^2^ on belatacept, and decreased from 52.5 to 44.9 on cyclosporin A. In the study by Budde et al., mean eGFR increased by 5.2 ml/min/1.73 m^2^ on belatacept and decreased by 1.9 on CNI.

Belatacept was also associated with a decrease in the risk to develop *de novo* donor specific antibodies (DSA). In BENEFIT, 4.6% of patients on belatacept developed *de novo* DSA during follow-up vs. 17.8% on cyclosporin A. In the study by Budde et al., 1% of patients switched to belatacept developed *de novo* DSA during follow-up vs. 7% on tacrolimus.

These results must be interpreted with caution as they do not necessarily mean that, on the long term, there would be differences on hard, clinically pertinent outcomes (such as graft loss or death). Indeed, eGFR slopes and *de novo* DSA are determined on the subgroup of patients alive, with a functioning graft and with available data, notably excluding patients that lost their graft after a rejection (an event that is probably more frequent with belatacept), thus creating a potential differential bias. Furthermore, since rejections are more frequent with belatacept than with CNI, one cannot exclude that, on the long term, more patients would lose their grafts because of a rejection occurring on belatacept than because of a CNI-mediated nephrotoxicity.

Surrogate endpoints, developed to predict hard, clinically pertinent outcomes based on intermediate outcomes, have been validated in kidney transplantation (19). As they integrate various parameters (e.g., eGFR, donor specific antibodies, biopsy findings, proteinuria), observed differences between groups on these integrative criteria seem more reliable than differences in a sole parameter (e.g., eGFR) to predict long-term outcomes, and could help reduce follow-up in clinical trials with no loss in statistical power. More data are needed on this important matter.

### Quality of Life

In a *post-hoc* study based on data from BENEFIT and BENEFIT-EXT, Dobbels et al. (20) found that belatacept, compared to cyclosporine, was associated with an increase in Physical Composite Scores at 3 years (49.2 vs. 47.1 in BENEFIT, 46.4 vs. 43.6 in BENEFIT-EXT, *p* < 0.05 for both comparisons) but not in Mental Composite Scores.

No study compared quality of life between patients receiving belatacept and tacrolimus.

## Non-Randomized Studies

Hereafter, we will review non-randomized clinical studies involving belatacept in KT. We will only consider studies that add significant contribution to data from RCTs, either because they strongly comfort their findings or because they make new ones. We will distinguish between comparative (i.e., where there is a control group of CNI-treated patients) and non-comparative studies. Among comparative studies, we will distinguish between those providing an adjusted analysis (i.e., with statistical methods to consider selection bias between groups) and those providing none (i.e., crude comparison between groups).

### Comparative Studies, Adjusted Analyses

In a registry study of 50 244 *de novo* KTRs in the US, 458 of whom received belatacept, Wen et al. (21) found that belatacept was associated with a 2.36 times increase in adjusted hazard of rejection during a 1-year follow-up, compared with tacrolimus. There was a decrease in risk for new onset diabetes on belatacept (3.8% vs. 2.2%). There were no significant differences in risk for death, loss of allograft function, PTLD or cancer.

In a propensity-matched registry study on 657 *de novo* KTRs treated with belatacept in the US, and on 3 210 controls on tacrolimus, Cohen et al. (22) found that belatacept was associated with a 3.12 times higher odds of rejection during the first year following KT. During a maximal follow-up of 8 years, there were no differences in risk for death or loss of allograft function between belatacept and tacrolimus.

In a propensity-matched cohort study of 181 KTRs switched to belatacept in Paris, France, and on 181 controls on CNI, Chavarot et al. (23) found that belatacept was associated with a 6.3 times increase in risk of CMV-disease during follow-up (17.7% vs. 2.8%). Most CMV diseases on belatacept were atypical, late onset, had gastrointestinal involvement, and 10% (4/40) were life-threatening.

In a cohort study of 609 KTRs in the US, 24 of which were receiving belatacept, Ou et al. (24) found, in a weighted analysis, that belatacept was associated with a 16.7-fold lower odds of responding to anti-SARS-CoV2 mRNA-based vaccination, compared to comparable patients not receiving belatacept. Overall, after two doses, 5% of patients to belatacept responded to vaccination, compared to 50% in comparable patients not receiving belatacept.

In a cohort study of 563 KTRs in Berlin, Germany, 45 of which were receiving belatacept, Liefeld et al. (25) found, in a multivariate analysis, that belatacept was associated with an absence of response to anti-SARS-CoV2 mRNA-based vaccine. Specifically, none of the patients receiving belatacept showed a seroconversion after two doses of vaccine, vs. 24% of patients on tacrolimus.

### Comparative Studies, Unadjusted Analyses

In a cohort study of 11 453 *de novo* KTRs in São Paulo, Brazil, 34 of whom received belatacept, Viana et al. (26) found that belatacept-treated patients had the highest risk of developing tuberculosis during follow-up (14.7% vs. 1.6% among patients receiving CNI; unadjusted HR 13.14 [95%CI: 5.3-32.8]).

In a cohort study of 168 *de novo* KTRs in Atlanta, 104 of whom were treated by belatacept, Karadkhele et al. (27) found that the risk for CMV viremia was higher on belatacept than on tacrolimus during a 2-year follow-up (50% vs. 34.4%, *p* = 0.047). Of note, all patients were CMV-seronegative patients receiving kidneys from CMV-seropositive patients, and all received valganciclovir in primary prophylaxis for 6 months following KT. Among patients that developed CMV viremia, the rate of resistance to ganciclovir was higher on belatacept than on tacrolimus (21.1% vs. 1.6%, *p* < 0.001).

In a cohort study on 49 *de novo* KTRs treated with belatacept in France, and on 74 controls treated with tacrolimus, Leibler et al. (28) found that the risk of acute T-cell mediated rejection was higher on belatacept during a 1-year follow-up (25.4% vs. 5.6%, *p* = 0.003). There was no difference in the risk for acute antibody mediated rejection. Of note, all patients had pre-formed donor specific antibodies (median fluorescence intensity 500 to 3000), received thymoglobulin as an induction therapy and had protocol biopsies at 3 months and 12 months.

In a cohort study of 60 *de novo* KTRs treated with belatacept in Atlanta, USA, and on 44 controls treated with tacrolimus, Parsons et al. (29) found that belatacept was associated with a significant reduction in cPRA as compared with tacrolimus. All patients had calculated panel reactive antibody (cPRA) higher than 97% and no DSA. Of note, this reduction was predominantly due to a decrease on the strength of anti-HLA class I antibodies.

### Non-comparative Studies

In a cohort study of 453 KTRs switched from CNI to belatacept in France, Bertrand et al. (30) found that opportunistic infections developed in 43 patients (9.3%) post-conversion, during a mean follow-up of 20.1 months. The risk for opportunistic infections was of 6.5 per 100 person-year (among which CMV disease: 2.8 per 100 person-year; Pneumocystis pneumonia: 1.6 per 100 person-year). Two patients developed PTLD, two patients developed JC virus infection with neurological symptoms, no patients developed BK virus nephropathy. At 1-year post-conversion, 22/453 (4.8%) patients died with a functioning graft, 42/453 (9.3%) were alive with a non-functioning graft, 24/453 (5.3%) had experienced a rejection.

In a cohort study of 103 KTRs switched from CNI to belatacept in Grenoble, France, Terrec et al. (31) found that glycated hemoglobin A1c (HbA1c) levels decreased from 6.2% pre-switch to 5.8% after 6 months of treatment (*p* < 0.001). Overall, beneficial effects on glycemic control were found whether patients had preexisting diabetes at the time of conversion or not.

### Belatacept for Non-kidney Solid Organ Transplant Recipients

In an international randomized controlled study of 260 liver transplant recipients, 153 of whom received belatacept, Klintmalm et al. (32) found that belatacept was associated with an increase in risk for a composite outcome (rejection, graft loss or death) within 6 months following transplantation. In an extended follow-up, two patients on belatacept developed PTLD, and an increase in mortality was noted in the subgroup of patients receiving belatacept at high dose. The study was then stopped.

In a randomized controlled study of 27 lung transplant recipients in the US, 13 of whom received belatacept, Huang et al. (33) found that belatacept was associated with an increase in risk of death following transplantation, as compared with CNI (3/13 death in the belatacept group, vs. 0/14 in the CNI group). The study was prematurely stopped.

In a randomized controlled study of 43 kidney-pancreas transplant recipients in the US, 22 of whom received belatacept, Stock et al. (15) found that belatacept was associated with an increase in risk of pancreas rejection (5/22 patients on belatacept vs. 1/21 patients on CNI). Among patients on belatacept, 1/22 patients died (vs. none on CNI) and 2/22 patients had a partial or total loss of pancreatic function (vs. none on CNI). The study was prematurely stopped.

In a multicenter retrospective non-comparative cohort study of 40 heart transplant recipients in France switched from CNI to belatacept, mainly due to impaired renal function on CNI, Launay et al. (34) found that belatacept was associated with a high rate discontinuation and adverse effects. At the end of follow-up, 4/40 (10%) of patients had died (2 of fatal rejection, 1 of invasive infection, 1 of non-compliance). Discontinuation rate was of 16/40 (40%). Most patients had an increase in eGFR after conversion, but one patient started renal replacement therapy despite CNI withdrawal.

Based on these studies, the increase in risk for rejection with belatacept seems more problematic in non-kidney solid organ transplantations than in kidney transplantation, with less clear benefits on renal function. So far, belatacept use in daily practice is restricted to few patients with very specific indications (35).

## Synhtesis

A synthesis of the current state of the medical literature on belatacept in kidney transplant recipients is provided in Table 2.

**Table 2:** Current evidence, based on data from comparative studies, on the benefits to use belatacept instead of tacrolimus for kidney transplant recipients.

	***De novo* KTRs**	**Switch from tacrolimus**
**Hard, clinically pertinent outcomes**		
Death with a functioning graft	No proven benefit vs. tacrolimus	
Loss of graft function	No proven benefit vs. tacrolimus	
Rejections	Higher risk with belatacept than with tacrolimus (Mostly T-cell mediated, mostly within a year after initiation)	
Cardiovascular events	No proven benefit vs. tacrolimus	
Infectious events	No proven benefit vs. tacrolimus	Higher risk for CMV disease with belatacept
Cancers	No proven benefit vs. tacrolimus	
**Surrogate endpoints**		
Estimated GFR	Higher estimated GFR with belatacept than with tacrolimus	
Donor specific antibodies	Less *de novo* DSA with belatacept than with tacrolimus	
Glycemic control	Better glycemic control with belatacept than with tacrolimus	

*GFR, glomerular filtration rate; DSA, donor specific antibodies; KTR, kidney transplant recipient*.

## Conclusion

Belatacept is a non-nephrotoxic non-diabetogenic immunosuppressive drug developed to increase graft survival, as compared to the current gold standard, tacrolimus—which is a nephrotoxic and diabetogenic drug. Despite proven beneficial effects of belatacept on glomerular filtration rate, glycemic control, and the appearance of de novo donor specific antibodies, there are, currently, no truly evidence-based benefits on renal graft survival, as compared with tacrolimus.

This can be the consequence of insufficiently powered studies to detect a better graft survival. On the other end, a lack of effect of belatacept is also possible since its benefits (absence of nephrotoxicity, less donor specific antibodies) may be undermined by adverse effects (higher rate of rejection and of CMV disease) which impair graft and patient survival.

For *de novo* kidney transplant recipients, the current state of the medical literature does not support the use of belatacept instead of tacrolimus. This is a direct consequence of the lack of RCTs with enough statistical power to compare belatacept against tacrolimus, in this setting, on hard, clinically pertinent outcomes. Such trials are urgently needed.

For stable kidney transplant recipients already receiving tacrolimus, additional RCTs and long-term follow-up of previous trials are needed to determine whether the observed differences in surrogate endpoints favoring belatacept (better eGFR, less *de novo* DSA, better glycemic control) will result in differences on clinically pertinent outcomes (death, loss of allograft function).

Belatacept is associated with an increase in risk for rejection, especially within the first year after treatment initiation. There is evidence that belatacept is associated with an increase in risk for CMV-disease. Due to this increased risk, patients on belatacept should be closely monitored, especially within the first year after initiation. Belatacept is associated with a reduced response rate to anti-SARS-CoV2 mRNA-based vaccination.

Most patients included in control groups in RCTs on belatacept received cyclosporin A, which is not the current gold standard for KTRs. Consequently, belatacept has not been routinely used in KTRs since its approval. Therefore, since few KTRs received belatacept since its approval, data from observational post-approval studies are of poor quality, with small sample sizes and are mostly non comparative, adding very few significant information.

As of 2022, most questions on belatacept (and all the important ones) are unanswered from an evidence-based medicine perspective. RCTs using tacrolimus as a comparator with long term follow up are essential to definitively establish the true benefits of belatacept in kidney transplantation.

## Data Availability Statement

The original contributions presented in the study are included in the article/supplementary material, further inquiries can be directed to the corresponding author.

## Author Contributions

YL: methodology, conceptualization, data curation, visualization, investigation, and writing–original draft. HF: project administration, supervision, methodology, conceptualization, investigation, and writing–review and editing. Both authors contributed to the article and approved the submitted version.

## Conflict of Interest

The authors declare that the research was conducted in the absence of any commercial or financial relationships that could be construed as a potential conflict of interest.

## Publisher's Note

All claims expressed in this article are solely those of the authors and do not necessarily represent those of their affiliated organizations, or those of the publisher, the editors and the reviewers. Any product that may be evaluated in this article, or claim that may be made by its manufacturer, is not guaranteed or endorsed by the publisher.
